# Inhalation injury: epidemiology, pathology, treatment strategies

**DOI:** 10.1186/1757-7241-21-31

**Published:** 2013-04-19

**Authors:** David J Dries, Frederick W Endorf

**Affiliations:** 1 Department of Surgery, Regions Hospital, 640 Jackson Street, St. Paul, MN, 55101, USA; 2 The Burn Center, Regions Hospital, 640 Jackson Street, St. Paul, MN, 55101, USA

**Keywords:** Smoke inhalation, Burns, Carbon monoxide, Cyanide, Bronchoscopy

## Abstract

Lung injury resulting from inhalation of smoke or chemical products of combustion continues to be associated with significant morbidity and mortality. Combined with cutaneous burns, inhalation injury increases fluid resuscitation requirements, incidence of pulmonary complications and overall mortality of thermal injury. While many products and techniques have been developed to manage cutaneous thermal trauma, relatively few diagnosis-specific therapeutic options have been identified for patients with inhalation injury. Several factors explain slower progress for improvement in management of patients with inhalation injury. Inhalation injury is a more complex clinical problem. Burned cutaneous tissue may be excised and replaced with skin grafts. Injured pulmonary tissue must be protected from secondary injury due to resuscitation, mechanical ventilation and infection while host repair mechanisms receive appropriate support. Many of the consequences of smoke inhalation result from an inflammatory response involving mediators whose number and role remain incompletely understood despite improved tools for processing of clinical material. Improvements in mortality from inhalation injury are mostly due to widespread improvements in critical care rather than focused interventions for smoke inhalation.

Morbidity associated with inhalation injury is produced by heat exposure and inhaled toxins. Management of toxin exposure in smoke inhalation remains controversial, particularly as related to carbon monoxide and cyanide. Hyperbaric oxygen treatment has been evaluated in multiple trials to manage neurologic sequelae of carbon monoxide exposure. Unfortunately, data to date do not support application of hyperbaric oxygen in this population outside the context of clinical trials. Cyanide is another toxin produced by combustion of natural or synthetic materials. A number of antidote strategies have been evaluated to address tissue hypoxia associated with cyanide exposure. Data from European centers supports application of specific antidotes for cyanide toxicity. Consistent international support for this therapy is lacking. Even diagnostic criteria are not consistently applied though bronchoscopy is one diagnostic and therapeutic tool. Medical strategies under investigation for specific treatment of smoke inhalation include beta-agonists, pulmonary blood flow modifiers, anticoagulants and antiinflammatory strategies. Until the value of these and other approaches is confirmed, however, the clinical approach to inhalation injury is supportive.

## Introduction

Respiratory injury resulting from inhalation of smoke or chemical products of combustion is associated with significant morbidity and mortality. Even in isolation, inhalation injury can be associated with longstanding pulmonary dysfunction [1]. Combined with cutaneous burns, inhalation injury increases fluid resuscitation requirements, incidence of pulmonary complications and overall mortality of thermal injury. Unfortunately, a consistent diagnostic strategy is unavailable and treatment is largely supportive [2-4]. We will review pathology, diagnostic options and medication strategies.

**Figure 1:**
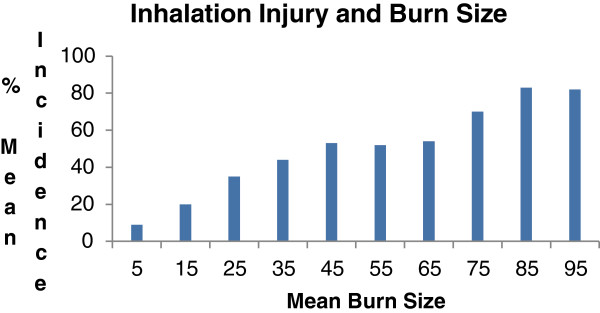
**Relationship between burn size and incidence of inhalation injury illustrates the rise in occurrence of inhalation injury with increasing burn size [**[5]**].**

**Figure 2:**
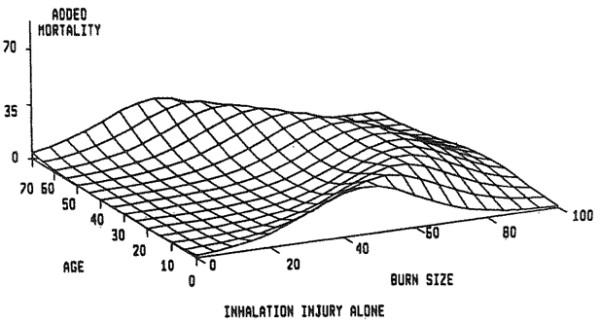
**Burn size as percentage of total body surface area on *X *axis, age on *Y *axis, and percent increment in mortality due to the presence of inhalation injury on *Z *axis are shown.** Mortality, in the presence of inhalation injury alone, rose by a maximum of approximately 20% in patients in midrange of severity of injury as indexed by age and burn size [5].

**Figure 3:**
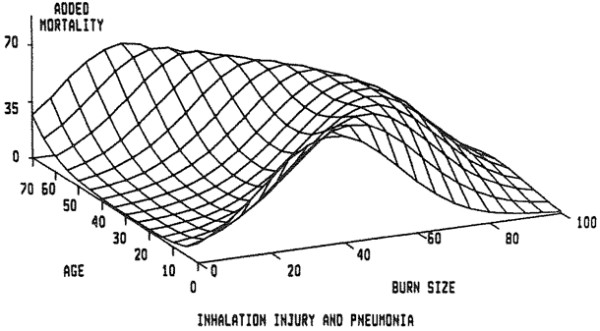
**Burn size as percentage of total body surface area on *X *axis, age on *Y *axis, and percent increment in mortality on *Z *axis are shown.** Mortality rose by a maximum of approximately 60% in patients in midrange of age and burn size when both inhalation injury and pneumonia were present [5].

The classic paper describing the effects of inhalation injury, and its principle complication, pneumonia, on mortality in burn patients comes from Shirani, Pruitt, Mason, and the U.S. Army Institute of Surgical Research in San Antonio, Texas [5]. A review of over 1,000 patients was conducted, in which data were gathered on status of inhalation injury on admission and development of pneumonia during hospitalization. Patients at risk for inhalation injury were investigated by bronchoscopy, Xenon lung scans, or both. The diagnosis of inhalation injury was made in 373 patients. With increasing burn size, there was a corresponding rise in the incidence of inhalation injury. The diagnosis of pneumonia was made at approximately 10 days for patients experiencing this complication along with inhalation injury. Three dimensional plots were constructed to demonstrate the incremental mortality of inhalation injury and inhalation injury when complicated by pneumonia on patients in this population. Expected mortality increased by a maximum of 20% in the presence of inhalation injury alone and 60% when both inhalation injury and pneumonia were present. The contributions of inhalation injury and pneumonia to mortality were found to be independent and additive. Expected mortality in patients with very small or very large burns was not affected by these pulmonary complications except at the extremes of age (Figures 1, 2 and 3).

Two other papers support the observations of Shirani and coworkers. A more recent meta-analysis on prognostic factors in burn injury with smoke inhalation reveals that overall mortality increased dramatically with inhalation injury (27.6% versus 13.9%). Extent of burn size and age were predictive of mortality. Another study included a predictive model of outcome with cutaneous injury plus smoke inhalation. In a review of 110 patients, percent Total Body Surface Area (TBSA) cutaneous injury, age and PaO_2_/FiO_2_ ratio were mortality predictors [6-8].

While many products and techniques have been developed to manage cutaneous injury, relatively few diagnosis-specific therapeutic options have been identified for patients with inhalation injury. Improvements in mortality from inhalation injury are mostly due to widespread improvements in critical care rather than focused interventions for smoke inhalation. In fact, one consensus statement indicates that treatment of inhalation injury has not kept pace with improvements in the care of cutaneous burns [9].

A variety of factors explain slower progress for improvement in management of inhalation injury. Burned cutaneous tissue may be excised and replaced with skin grafts, but njured pulmonary tissue must merely be supported and protected from secondary injury. The critically ill burn patient has multiple mechanisms in addition to smoke inhalation that may contribute to lung injury such as sepsis, Ventilator-Induced Lung Injury (VILI) or a systemic inflammation in response to burns. Thus, inhalation injury has a significant effect on burn patient outcome but is difficult to separate from the contribution of other mechanisms which also affect the lungs [2,10,11].

A significant limitation for clinicians studying smoke inhalation has been the lack of uniform criteria for diagnosis of inhalation injury, scaling its severity and identifying a common terminology to describe outcomes [2,9]. Thus, comparative studies are difficult to evaluate. Some practitioners describe patients requiring intubation and mechanical ventilation after smoke inhalation. Other studies emphasize nuclear medicine scans for the metabolic diagnosis of inhalation injury. Multicenter trials have the confounding impact of differing local definitions of inhalation injury. The need for standardized diagnostic criteria and a quantifying system for inhalation injury have been recognized in the burn literature for many years.

## Anatomy and physiology of inhalation injury

Inhalation injury may describe pulmonary trauma caused by inhalation of thermal or chemical irritants. Anatomically, injuries are divided into three classes: 1) heat injury which is restricted to upper airway structures except in the case of steam jet exposure, 2) local chemical irritation throughout the respiratory tract and 3) systemic toxicity as may occur with inhalation of carbon monoxide or cyanide [3].

### Heat injury to the upper airway

Air temperature in a room containing a fire reaches 1000°F. Because of the combination of efficient heat dissipation in the upper airway, low heat capacity of air and reflex closure of the larynx, super-heated air usually causes injury only to airway structures above the carina. Injury to these airway structures may cause massive swelling of the tongue, epiglottis, and aryeepiglottic folds with obstruction. Airway swelling develops over a matter of hours as fluid resuscitation is ongoing. Initial evaluation is not a good indicator of the severity of obstruction that may occur later [3,12].

Respiratory status must be continuously monitored to assess the need for airway control and ventilator support. If history and initial examination cause suspicion of significant thermal injury to the upper airway, intubation for airway protection should be considered.

### Chemical injury to the lower airway

Most substances when burned, generate material toxic to the respiratory tract [2,3,9]. Burning rubber and plastic produces sulfur dioxide, nitrogen dioxide, ammonia and chlorine with strong acids and alkali when combined with water in the airways and alveoli. Laminated furniture contains glues and wall paneling also may release cyanide gas when burned. Burning cotton or wool produces toxic aldehydes. Smoke-related toxins damage epithelial and capillary endothelial cells of the airway. Histologic changes resemble tracheobronchitis. Mucociliary transport is destroyed and bacterial clearance reduced. Alveolar collapse and atelectasis occur due to surfactant loss. Alveolar macrophages are stressed leading to inflammatory response with chemotaxins. Early inflammatory changes occurring in the airway are followed by a period of diffuse exudate formation. Bronchiolar edema may become severe. A combination of necrotizing bronchitis, bronchial swelling, and bronchospasm causes obstruction of large and small airways. Wheezing occurs with bronchial swelling and irritant receptor stimulation. Increased capillary permeability magnifies airway and pulmonary edema [13-15].

Respiratory failure may occur from 12 to 48 hours after smoke exposure. Characteristics are decreased lung compliance, increased ventilation perfusion mismatch, and increase in dead space ventilation. Injury may progress to mucosal sloughing and intrapulmonary hemorrhage with mechanical obstruction of lower airways and flooding of alveoli [16,17]. Because of necrosis of respiratory epithelium, patients are predisposed to secondary bacterial invasion and superimposed bacterial pneumonia [5]. Recovery may require several months [18].

### Carbon monoxide and cyanide exposure

Carbon monoxide is an odorless, tasteless, nonirritating gas produced by incomplete combustion. Carbon monoxide poisoning is a major source of early morbidity in burn-injured patients with many fatalities occurring at the scene of the fire due to this mechanism. Carboxyhemoglobin levels exceed 10% in a closed space fire. Significant injury may occur in a short period of time with the exposure with as little as 10% carboxyhemoglobin [3,19].

**Figure 4:**
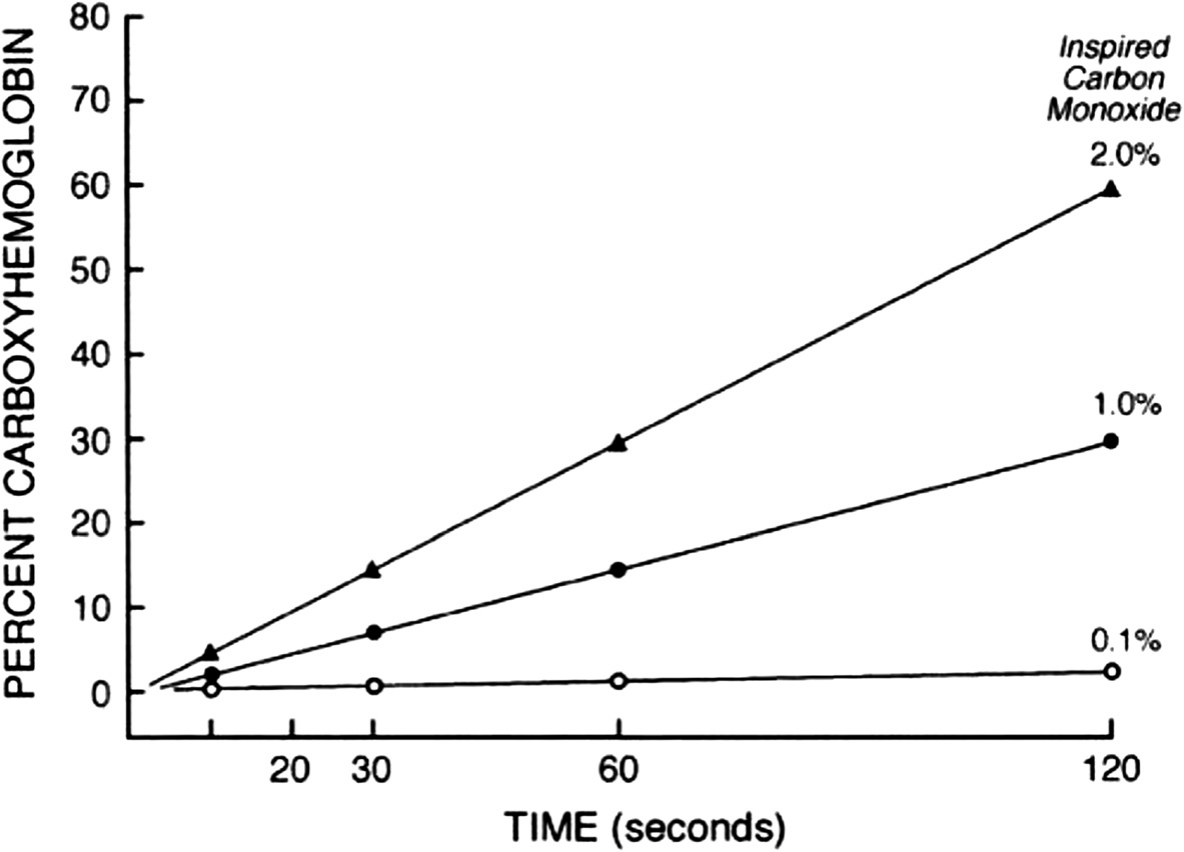
**Hemoglobin is converted rapidly to carboxyhemoglobin in the presence of carbon monoxide [**[3]**].**

**Figure 5:**
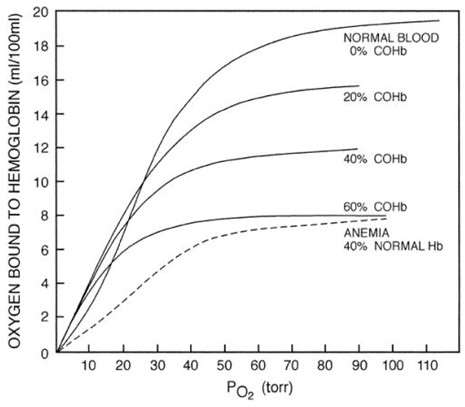
**Carboxyhemoglobin-induced changes in the oxygen-hemoglobin dissociation curve.** Oxygen-carrying capacity is markedly diminished when carboxyhemoglobin values reach 40% to 50%. In addition, the leftward displacement of the oxygen-hemoglobin dissociation curve makes the oxygen that is bound to hemoglobin less available for delivery to tissues [3].

The affinity of carbon monoxide for hemoglobin is 200 times greater than for oxygen. Carbon monoxide competes with oxygen for hemoglobin binding which shifts the oxyhemoglobin dissociation curve to the left and alters its shape. Oxygen delivery to tissues is compromised because of reduced oxygen carrying capacity of the blood and less efficient dissociation at the tissue level. Carbon monoxide competitively inhibits intracellular cytochrome oxidase enzyme systems, most notably cytochrome P-450 resulting in inability of cellular systems to utilize oxygen (Figures 4 and 5) [20,21].

Inhaled hydrogen cyanide, produced during combustion of multiple household materials, also inhibits the cytochrome oxidase system and may have a synergistic effect with carbon monoxide producing tissue hypoxia and acidosis as well as a decrease in cerebral oxygen consumption [3,21].

Carbon monoxide poisoning may be difficult to detect. The absorbent spectrum of carboxyhemoglobin and oxyhemoglobin are very similar and pulse oximeters cannot distinguish between the two forms of hemoglobin. The PaO_2_ measure from an arterial blood gas reflects the amount of oxygen dissolved in plasma but does not quantitate hemoglobin saturation, the most important determinant of oxygen carrying capacity of the blood. Carboxyhemoglobin levels may be measured directly but this test is rarely available at the incident scene. Because of the inevitable delay between smoke exposure and carboxyhemoglobin testing, levels measured on arrival at a healthcare facility do not reflect the true extent of intoxication [3,22,23].

Half-life of carboxyhemoglobin is 250 minutes for the victim breathing room air. This is reduced to 40 to 60 minutes with inhalation of 100% oxygen [3,15]. While hyperbaric oxygenation will further reduce the half-life of carboxyhemoglobin, the hyperbaric chamber is a difficult environment in which to monitor the patient, perform fluid resuscitation, and provide initial burn care. Patients with the greatest need for hyperbaric oxygen therapy are frequently the most difficult to manage in this environment [24].

## Diagnosis of inhalation injury

For the clinician, the diagnosis of inhalation injury is a somewhat subjective decision based largely on a history of smoke exposure in a closed space. Physical findings including facial injury, singed nasal hairs, soot in the proximal airways, carbonaceous sputum production and changes in voice may help support the diagnosis [2,3,9,22]. These findings may be confirmed by diagnostic studies including fiberoptic bronchoscopy, typically performed within 24 hours of admission [25]. History includes mechanisms of exposure such as flame, electricity, blast injury, steam or hot liquid, quality of inhaled irritants (*house fire or industrial toxins*) and duration of exposure with further complications caused by loss of consciousness or physical disability. Physical examination may include findings such as visible injury to the respiratory tract, airway edema or evidence of pulmonary parenchymal damage and dysfunction.

Diagnostic criteria for inhalation injury are complicated by heterogeneous presentation and distinguishing between exposure to inhaled irritants and injury based on heated gas exposure [9,26]. Progressive respiratory failure may not be directly proportional to the degree of smoke exposure. Such differences are likely due to composition of inhaled materials and differences in host response.

Multiple burn centers have demonstrated that patients with inhalation and burn injuries require increased fluid volumes during immediate resuscitation when compared to individuals with burn injury alone [4,9,27]. Changes in lung compliance and airway resistance have also been proposed as predictors of outcome and scales for severity of inhalation injury. Scoring systems, based on bronchoscopic evaluation, have been used for inhalation injury and attempts to identify the relationship of this data to the development of Acute Respiratory Distress Syndrome have been made. Endorf and Gamelli, in recent work, examine the degree of inhalation injury, PaO_2_/FiO_2_ ratio, and effects on fluid requirements during acute resuscitation. Table 1 demonstrates a typical set of bronchoscopic criteria for grading of inhalation injury [25].

**Table 1:** Bronchoscopic criteria used to grade inhalation injury

	
**Grade 0 (No Injury):**	**Absence of carbonaceous deposits, erythema, edema, bronchorrhea, or obstruction.**
**Grade 1 (Mild Injury):**	**Minor or patchy areas of erythema, carbonaceous deposits in proximal or distal bronchi. [any or combination]**
**Grade 2 (Moderate Injury):**	**Moderate degree of erythema, carbonaceous deposits, bronchorrhea, with or without compromise of the bronchi.**
	**[any or combination]**
**Grade 3 (Severe Injury):**	**Severe inflammation with friability, copious carbonaceous deposits, bronchorrhea, bronchial obstruction.**
	**[any or combination]**
**Grade 4 (Massive Injury):**	**Evidence of mucosal sloughing, necrosis, endoluminal obliteration. [any or combination]**

**Endorf and Gamelli [**[25]**].**

*Reproduced with permission from J Burn Care Res and Endorf, et al.*

These workers reviewed 80 patients from a single center with suspected inhalation injury requiring intubation, mechanical ventilation, and fiberoptic bronchoscopy during the first 24 hours of hospitalization. Details of burn injury were collected and patients categorized according to a bronchoscopic grading system. Pulmonary mechanics and gas exchange were examined at regular intervals including lung compliance and PaO_2_/FiO_2_ ratio. Total fluid volume infused was noted for the first 48 hours after burn injury [25].

Patients with more severe bronchoscopic injury on initial bronchoscopy (Grades 2, 3, 4) had significantly worse survival than patients with bronchoscopic Grades 0 or 1 (*p* = 0.03). Contrary to reports of other investigators, these workers noted that high-grade bronchoscopic findings were not associated with increased fluid requirements. Initial pulmonary compliance also did not correlate with acute fluid requirements. Notably, patients with a PaO_2_/FiO_2_ ratio <350 at presentation had a statistically significant increase in fluid resuscitation requirement compared with patients having a PaO_2_/FiO_2_ ratio >350 (*p* = 0.03) (Tables 2 and 3).

**Table 2:** Comparison for bronchoscopic grade of inhalation injury

	**Group 1**	**Group 2**	***P *Value**
	**(Grades 0 and 1)**	**(Grades 2, 3, 4)**	
	**25 Patients**	**35 Patients**	
**mL/kg/%TBSA**	**6.6 (±0.7)**	**6.7 (±0.4)**	**.88**
**Ventilator days**	**8.6 (±1.4)**	**12.8 (±2.2)**	**.11**
**Survival**	**21 (84%)**	**20 (57%)**	**.03**
**Initial compliance**	**49.9 (±4.4)**	**49.7 (±3.1)**	**.98**
**Initial P:F Ratio**	**371.5 (±32)**	**329.7 (±29)**	**.33**

**Endorf and Gamelli [**[25]**].**

*Reproduced with permission from J Burn Care Res and Endorf, et al.*

**Table 3:** Comparison by P:F ratio

	**P:F <350**	**P:F >350**	***P *Value**
	**(30 Patients)**	**(30 Patients)**	
**mL/kg/%TBSA**	**7.4 (±0.4)**	**5.9 (±0.5)**	**.03**
**Ventilator days**	**12.2 (±2.4)**	**0.9 (±1.5)**	**.21**
**Survival**	**18 (60%)**	**23 (77%)**	**.17**

**Endorf and Gamelli [**[25]**].**

*Reproduced with permission from J Burn Care Res and Endorf, et al.*

Most writers agree that a consensus regarding the diagnosis of inhalation injury will be based on modalities which are widely available and do not require highly specialized skills. A consistent vocabulary for description of injury and its physiologic effects is also required along with reliable description of the composition and disposition of inhaled irritants with some grading of intensity of exposure [28].

The best tools presently available for diagnosis of inhalation injury are clinical presentation and bronchoscopic findings. Difficulty comes with attempts to predict which patients are vulnerable to resuscitation complications, increased pulmonary dysfunction, respiratory failure and mortality. Attempts to identify prognostic factors for patients with smoke inhalation have been made. It has been difficult to identify reliable indicators of progressive respiratory failure in patients with smoke inhalation. Moreover, proximal injury observed by bronchoscopy is frequently greater than peripheral pulmonary parenchymal injury. Several investigative teams show lack of correlation between severity of bronchoscopic findings, fluid resuscitation requirements, development of Acute Respiratory Distress Syndrome (ARDS) and other clinical outcomes [25,28-31]. Other diagnostic modalities such as 99-technetium scanning and xenon scanning may confirm inhalation injury but due to logistical reasons are not widely used in the initial evaluation of smoke inhalation [32].

## Treatment strategies

### Bronchoscopy

In many centers, bronchoscopy has a role limited to obtaining lavage fluid for culture and assessing the degree of airway injury which may predict outcome [33]. Severe inhalation injury is in part a mechanical process characterized by pulmonary edema, bronchial edema, and secretions, can occlude the airway leading to atelectasis and pneumonia. Aggressive use of bronchoscopy is highly effective in removing foreign particles and accumulated secretions that worsen the inflammatory response and may impede ventilation [34,35]. While it seems intuitive that bronchoscopy could improve pulmonary hygiene and outcomes by removing secretions and epithelial slough in burn patients, only recently has this question been addressed by a review of the National Burn Repository of the *American Burn Association*[33].

Carr and coworkers reviewed the National Burn Repository from 1998 to 2007 to determine outcome differences in burn patients with inhalation injury and pneumonia who did and did not receive bronchoscopy [33]. Patients with a 30-59% Total Body Surface Area burn and pneumonia who underwent bronchoscopy had a decreased duration of mechanical ventilation compared to patients who did not have bronchoscopy. Patients with larger injuries and pneumonia did not have improved outcomes with bronchoscopy. When patients having at least one bronchoscopy procedure were compared with those who did not undergo bronchoscopy, the patients receiving this test had a shorter length of intensive care unit and hospital stay. Hospital charges were higher in patients who did not undergo bronchoscopy compared with those who received this procedure. When compared with patients who did not undergo bronchoscopy, patients who did have one or more bronchoscopic procedures had a reduced risk of death by 18%. However, while strong trends were present, the mortality benefit associated with bronchoscopy and the reduction in hospital cost represented trends which did not reach statistical significance.

### Carbon monoxide toxicity

Morbidity and mortality associated with carbon monoxide toxicity are the result of hypoxic states associated with interference with oxygen transport at the cellular level and compromise of electron transport within cells. Other potential mechanisms include binding to myoglobin or hepatic cytochromes and peroxidation of cerebral lipids. The extent of injury is dependent on the concentration of carbon monoxide, duration of exposure and underlying health status of the exposed individual [36,37].

Short- and long-term morbidity of carbon monoxide toxicity involves neurologic and vascular consequences. Neurologic sequelae are divided into two syndromes: 1) persistent neurologic sequelae and 2) delayed neurologic sequelae. Persistent neurologic sequelae involve neurologic deficits occurring after carbon monoxide exposure that may improve over time. Delayed neurologic sequelae is a relapse of neurologic signs and symptoms after a transient period of improvement. Distinguishing between these conditions may be difficult. Symptoms of chronic carbon monoxide toxicity may include fatigue, affective conditions, emotional distress, memory deficits, difficulty working, sleep disturbances, vertigo, neuropathy, paresthesias, recurrent infections, polycythemia, abdominal pain and diarrhea [37-39].

Neuropsychological sequelae are common after carbon monoxide poisoning. In some trials, 40% of involved patients treated with normobaric oxygen had cognitive sequelae when evaluated six weeks after carbon monoxide exposure and a similar number had affective sequelae. Other potential consequences include gait and motor disturbances, peripheral neuropathy, hearing loss and vestibular abnormalities, dementia and psychosis. These changes may be permanent [37,40-42].

Immediate management of carbon monoxide toxicity is administration of normobaric oxygen by means of a nonrebreather reservoir facemask supplied with high flow oxygen or 100% oxygen by means of an artificial airway. Administration of normobaric oxygen hastens elimination of carbon monoxide but one trial did not show reduction in cognitive sequelae after inhalation of normobaric oxygen as compared with no supplemental oxygen therapy [36,37]. Since normobaric oxygen is safe, readily available and inexpensive, however, it should be provided until a carboxyhemoglobin level is less than 5%. Initial support of the exposed patient should emphasize adequate ventilation and perfusion, neurologic examination, exposure history and measurement of arterial blood gases by co-oximetry to assess gas exchange, metabolic status and carboxyhemoglobin level. A carboxyhemoglobin level greater than 3% in nonsmokers or greater than 10% in smokers confirms exposure to carbon monoxide. *The carbon monoxide level does not correlate with the presence or absence of initial symptoms or with later outcomes*[35,43,44].

Carbon monoxide exposure can exacerbate angina and cause cardiac injury even in persons with normal coronary arteries. Thus, exposed patients may require cardiovascular investigation including electrocardiogram and measurement of cardiac enzymes. If cardiac injury is present, cardiology consultation should be considered [37,45,46].

The use of hyperbaric oxygen has been advocated to treat carbon monoxide exposure under the hypothesis that rapid displacement of carbon monoxide from hemoglobin at 100% oxygen using hyperbaric pressures will reduce duration of the cellular hypoxic state [36,37]. Use of hyperbaric oxygen results in more rapid displacement of carbon monoxide. Absolute indications and outcomes for hyperbaric oxygen remain controversial because of lack of correlation between the only available diagnostic tool, carboxyhemoglobin levels, and the severity of the clinical state and outcomes of the initial insult or therapies [36]. In addition, there is no standard for duration or intensity of hyperbaric oxygen therapy. Hyperbaric oxygen has potential complications including barotrauma, tympanic membrane disruption, seizures and air embolism [47-50].

Among published clinical trials of hyperbaric oxygen therapy, few satisfy all consolidated standards for the reporting of trials guidelines including double-blinding, enrollment of all eligible patients, a priori definitions of outcomes and high rates of follow-up [37,49,51,52]. One single center prospective trial showed that the incidence of cognitive sequelae was lower among patients who underwent three hyperbaric oxygen sessions (initial session of 150 minutes, followed by two sessions of 120 minutes each, separated by an interval of 6 to 12 hours) within 24 hours after acute carbon monoxide poisoning than among patients treated with normobaric oxygen (25% versus 46%, p = 0.007 and p = 0.03 after adjustment for cerebellar dysfunction and stratification). Use of hyperbaric oxygen in this trial reduced the rate of cognitive sequelae at 12 months (18% versus 33% with normobaric oxygen; p = 0.04). This trial did not, however, clearly identify subgroups of patients in whom hyperbaric oxygen was more or less beneficial [37].

A Cochrane review of six trials including two published in abstract form did not support the use of hyperbaric oxygen for patients with carbon monoxide poisoning [53]. A more recent Cochrane review also failed to demonstrate convincing benefit from hyperbaric oxygen therapy [54]. However, multiple flaws in the reviewed trials were identified [36,37]. The use of hyperbaric oxygen therapy for carbon monoxide victims continues to be guided by standards of the community rather than scientific consensus.

Patients with carbon monoxide poisoning should be followed medically after discharge. Extent and rate of recovery after poisoning are variable and recovery is often complicated by sequelae which can persist after exposure or develop weeks after poisoning and which may be permanent. Specific therapy for sequelae after carbon monoxide exposure is not available. Patients with sequelae should have symptoms addressed through cognitive, psychiatric, vocational, speech, occupational and physical rehabilitation. Data on these interventions in patients with carbon monoxide sequelae are lacking [37,40].

An important trial examined long-term outcomes of patients with acute carbon monoxide poisoning [55]. Over 1,000 patients treated over a 30 year period were examined. Patients studied were treated with hyperbaric oxygen and survived the acute poisoning episode. Long-term mortality was compared to a standard population. Survivors of acute carbon monoxide poisoning experienced excess mortality in comparison to the general population. Excess mortality was highest in the group initially treated for intentional carbon monoxide poisoning. For the entire group, major causes of death were mental and psychiatric disorders, injuries and violence. Other more specific causes of death were alcoholism, motor vehicle crash with pedestrians, motor vehicle crashes of unspecified type, accidental poisoning and intentional self-harm. Consistent with data mentioned above, no difference in survival was observed by measure of carbon monoxide poisoning severity after controlling for age, gender, race and intent of carbon monoxide poisoning.

### Cyanide toxicity

Cyanide is produced by combustion of natural or synthetic household materials including synthetic polymers, polyacrylonitrile, paper, polyurethane, melamine, wool, horsehair and silk [56,57]. Cyanide can be detected in trace amounts in smoke at house fires and in the blood of smokers and fire victims. Ingestion of cyanide products produces metabolic acidosis which is also seen in burn patients during resuscitation. Cyanide is a normal human metabolite which the body can detoxify. Cyanide can be produced *in vitro* by normal human blood and *in situ* in certain organs after death. Much of the interest in cyanide as a toxin related to inhalation injury stems from the availability of a cyanide antidote kit.

Barillo recently reviewed the evidence regarding testing of smoke inhalation victims for cyanide [57,58]. Unfortunately, a simple and rapid blood assay for cyanide is lacking and may be of limited utility as cyanide is an intracellular toxin. As noted above, cyanide is a normal metabolite in humans and can be produced and degraded in blood samples *in vitro*. Erythrocytes convert thiocyanate to cyanide *in vitro* and because blood cyanide is mainly bound to erythrocytes, autolysis of red blood cells may elevate blood cyanide levels. In normal individuals, blood cyanide levels range from up to 0.3 mg/L in nonsmokers to 0.5 mg/L in smokers. Firefighters, despite chronic smoke exposure, have relatively normal blood cyanide levels. Cyanide is mildly elevated in both fire survivors and fire fatalities. Survival with blood cyanide levels of 7–9 mg/L has been documented after cyanide ingestion or inhalation. Recommendations for treatment of cyanide intoxication in smoke victims are extrapolated from limited industrial experience or from suicide and homicide victims. Overt cyanide poisoning is uncommon and little human data is available [57,59].

A popular cyanide antidote kit utilizes a series of reactions with oxidation of hemoglobin to methemoglobin which binds cyanide forming cyanomethemoglobin [60,61]. As cyanomethemoglobin dissociates, free cyanide is converted to thiocyanate by hepatic mitochondrial enzymes using colloidal sulfate or thiosulfate. Thiocyanate is then excreted in the urine. Despite popularity of the cyanide antidote kit, documented effectiveness is limited [57,58,62]. Notably, a methemoglobin level of 20-30% is required to optimally bind cyanide. Additionally, this is contraindicated in patients with concurrent carbon monoxide poisoning as the conversion of carboxyhemoglobin to methemoglobin may exacerbate hypoxia. Another management strategy utilizes sodium thiosulfate as a substrate in conversion of cyanide to thiocyanate and is reported to be an effective antidote when used with or without nitrite. Prospective trials utilizing this strategy are lacking apart from case studies. Administration at recommended doses is without serious side effects while nausea, retching and vomiting have been reported [57,63].

European data suggests treatment of cyanide poisoning with chelating agents such as dicobalt edetate or hydroxycobalamin. Dicobalt edetate is associated with anaphylaxis and can produce hypertension, rhythm changes or cobalt poisoning. At present, dicobalt edetate is not available in the United States. It has been used in Great Britain [57,64,65]. Hydroxycobalamin is an effective cyanide antidote at a dose of 100 mg/kg. Unfortunately, in the United States, hydroxycobalamin has been available at 1 mg/mL concentrations which limits usefulness as approximately 10 L of material would be needed to neutralize a fatal cyanide dose [58,66]. The European approach to cyanide poisoning is quite aggressive relative to the United States. In Europe, 1 mg/L blood cyanide level is considered significant or fatal. Hydroxycobalamin and dicobalt edetate are used together to manage cyanide exposure in France [58,65].

Cyanide antidotes have recently been reviewed by Hall and coworkers. Scattered investigators in the United States and French clinicians continue to study a variety of agents available for management of this problem. A number of agents are available with differing mechanisms of action. Most of the clinical work, originating from firefighters in Paris emphasizes the use of hydroxycobalamin in smoke inhalation victims with high risk smoke exposure. Various antidotes available for cyanide have varied tolerability and safety profiles. For example, dicobalt edetate use is limited by toxicity concerns. Another cyanide antidote used in Germany is 4-dimethylaminophenol. Like sodium nitrate and amyl nitrite, 4-dimethylaminophenol is thought to neutralize cyanide by inducing methemoglobin. Unfortunately, methemoglobin concentrations and toxicity can be significant with this agent. Use of dicobalt edetate is limited by cobalt toxicity. Of studied agents, hydroxycobalamin has the smallest toxicity profile apart from allergic reactions. Because of a favorable side effect profile, this agent has been used in small studies of prehospital and empiric treatment of smoke exposure. Hydroxycobalamin has rapid onset of action and neutralizes cyanide without interfering with cellular oxygen use. At present, multiple investigators suggest that if employed, hydroxycobalamin is the antidote of first resort in cyanide exposure [67,68].

Hydroxycobalamin therapy has been used to prevent cyanide toxicity in patients receiving intravenous nitroprusside and to treat toxic amblyopia and optic neuritis caused by cyanide in tobacco smoke. In these applications, hydroxycobalamin is generally well tolerated but may be associated with side effects of headache, allergic reactions, skin and urine discoloration, hypertension or reflex bradycardia [58,63,69]. Hyperbaric oxygen therapy for cyanide has also been advocated. There is little objective data to support this application [58,70,71]. In light of recent experience with hyperbaric oxygen in carbon monoxide toxicity, a role for this modality in cyanide exposure is questionable [54].

In summary, the need for specific antidotes in cyanide toxicity is unclear. Aggressive supportive therapy directed to restoration of cardiovascular function with provision of supplemental oxygen augments hepatic clearance of cyanide without specific antidotes and should be first line treatment. Even with severe cyanide poisoning (blood levels of 5–9 mg/L), after cyanide ingestion or smoke inhalation, survival has been documented with aggressive supportive therapy provided without cyanide antidotes [58,72,73]. Another critical issue is the lack of a rapid cyanide assay to document actual poisoning before antidote administration is considered. If an accurate and rapid cyanide assay is available, prospective studies can then be designed to address the efficacy of various treatment options.

## Mechanical ventilation

There is no ideal respiratory support strategy for the patient with inhalation injury. Consensus recommendations for mechanical ventilation continue to serve as general guidelines [74]. Ventilator strategies must support oxygenation and ventilation and reflect the experience of the clinical team managing the patient. Limitation of pressure, acceptance of permissive hypercapnia and strategies to manage secretions are important. A significant number of patients with smoke inhalation will develop pneumonia in association with mechanical ventilation. Routine prevention strategies include elevation of the head of the bed, frequent position changes and oral care. Antibiotic prophylaxis has no role and may increase infection rates. Extracorporeal membrane oxygenation is perhaps the most dramatic rescue therapy and clearly not applicable as a standard therapy at this time [75-77]. Simple strategies such as prone positioning are more practical in the hypoxic patient [78].

A number of ventilation modes have been recommended for specific application to the patient with burn injury. High Frequency Oscillatory Ventilation (HFOV) supports the lung at a mean airway pressure above that used in conventional ventilation. Oscillations may cause significant pressure swings in the endotracheal tube while pressure fluctuations are attenuated at the alveolar level. Small studies suggest modest improvement in oxygenation with HFOV over conventional ventilation strategies. Two recent major trials do not support widespread use of HFOV [79-81]. Airway Pressure Release Ventilation (APRV) uses continuous positive airway pressure applied at a high level with intermittent releases of airway pressure. Spontaneous breathing during APRV more closely mimics gas distribution of normal breathing as opposed to mechanically controlled breaths which produce a less physiologic gas distribution. APRV has been used in a variety of critically ill patients. A number of physiologic concerns remain to be addressed before widespread application of APRV can be recommended. For example, APRV can be associated with significant elevation in mean airway pressure while allowing lung collapse between episodes of continuous positive airway pressure. In patients with critical illness, spontaneous breathing through an open ventilator circuit may not be feasible. Finally, pulmonary transmural pressure in APRV is not controlled and can be elevated significantly. It appears that APRV can be used effectively by clinicians familiar with its rationale and experienced in its use. However, advantages of APRV over optimized conventional ventilation have not been demonstrated and its ultimate role for management of patients with respiratory failure has yet to be proven [82,83].

### Noninvasive ventilation

Many studies report benefit with noninvasive ventilation due to avoidance of endotracheal intubation and its associated complications. Without an endotracheal tube, patients communicate more effectively, require less sedation and are more comfortable. In addition, patients are able to continue with standard oral care. Trauma associated with endotracheal tube insertion is avoided along with sinusitis and impaired swallowing after extubation. The benefit of noninvasive ventilation most discussed in the literature is reduction in incidence, cost impact and subsequent mortality of pneumonia [84,85].

A key component of the success of noninvasive ventilation has been selection of awake, cooperative, spontaneously breathing patients. These individuals must be able to protect their airway. Hemodynamic or electrocardiographic instability or an unstable airway argue against the use of noninvasive ventilation. The unconscious patient with significant facial injuries is not a candidate for noninvasive ventilation. Further contraindications include compromised cough and the need for significant clearance of secretions. High secretion load and facial trauma are often seen with inhalation injury. Relative contraindications include inability to fit and seal masks and helmets secondary to injury or facial deformity including facial hair. Uncooperative patients or those who will not leave a mask in place, not cough when prompted or are unable to remove the mask in the event of emesis are not good candidates for noninvasive ventilation. If pressures used to ventilate the patient are maintained below 30 mmHg, the closing pressure of the lower esophageal sphincter should not be overcome and aerophagia should be relatively uncommon. Finally, morbid obesity is a relative contraindication due to increased ventilator pressure requirements arising from body habitus and weight of the chest wall or abdominal viscera with the patient in bed [84].

The optimal time to consider use of noninvasive ventilation in the burn injured patient is unclear. Historically, other patient groups have been treated with noninvasive ventilation when signs of hypoxemia or hypercarbia are present. Unlike other patient groups where respiratory compromise is generally progressive, the insult faced by the burn patient may be great in the initial hours after injury during high volume fluid resuscitation. During these initial hours, the risk of edema to burned and unburned tissue is signficiant. Noninvasive ventilation may be considered as a prophylactic strategy during resuscitation in high risk patients even before frank signs of respiratory insufficiency appear.

The most serious complication of noninvasive ventilation is failure to recognize when this therapy is not providing adequate ventilation, oxygenation or airway support. Delayed intubation may cause continued deterioration of the patient. Never lose a patient for failure to intubate [84,85].

### Ventilation

Patients with various forms of lung injury are now being treated with ventilator strategies involving limitation of minute ventilation through use of low tidal volumes resulting in a tendency toward hypercapnia. While hypercapnia in the setting of acute lung injury may be addressed in various ways, there is growing evidence that acceptance may be a better alternative than aggressive pursuit of normal carbon dioxide tension [86].

Airway pressures as low as 30 cmH_2_O have been associated with lung injury in animal models. This pressure corresponds with a normal static inflation pressure for total lung capacity in humans. Thus, maintaining plateau pressure <30 cmH_2_O is a reasonable approach to maintain aerated lung regions below normal maximum volume. This observation is important because high tidal volume ventilation may be insensitive to loss of lung volume available for gas exchange due to the effects of inhalation injury. Gattinoni and coworkers suggest that as little as 20% of the lung may be aerated in patients with severe respiratory failure. Thus, normal clinical tidal volumes and airway pressures may be dangerous [87-89].

At present, there is insufficient data to suggest that hypercapnia should be independently induced outside the context of a protective ventilation strategy. Ventilator strategies involving hypercapnia are acceptable within clinically reasonable hemodynamic bounds. Hypercapnic acidosis has been demonstrated to increase cardiac output in ARDS patients. Data from apnea tests and brain dead patients suggests tolerance of a pH to 7.2 and a PCO_2_ >75 without hemodynamic consequences. At greater degrees of hypercapnia and acidosis, hemodynamic instability may become a limiting factor [89-92].

### Oxygenation

Application of positive airway pressure is intended to replace or supplement respiratory muscle function and correct hypoxemia associated with alveolar hypoventilation. Reversal of hypoxemia caused by intrapulmonary shunt requires interventions that open lung units for gas exchange. In patients with lung edema, atelectasis or other injury, Positive End-Expiratory Pressure (PEEP) may increase arterial oxygenation by increasing functional residual capacity, reducing venous add mixture, shifting tidal volume to a more compliant portion of the pressure volume curve and preventing loss of lung compliance during mechanical ventilation. Work of breathing may also be reduced [89,93-95].

PEEP also has a value beyond maintaining airway patency. In patients with obstructive respiratory disease, lungs may fail to deflate to functional residual capacity at end expiration. Alveolar pressure remains positive in these individuals to an extent dependant on the volume of trapped air. This phenomenon is referred to as “auto-PEEP or extrinsic PEEP”. In the presence of auto-PEEP, application of external PEEP is beneficial during spontaneous breathing as respiratory work is reduced and during patient-triggered modes of ventilation where breath initiation is supported. Optimal administration of external PEEP in the setting of auto-PEEP reduces inspiratory muscle effort and improves patient ventilator interaction [96].

PEEP has hemodynamic effects as well. Increased intrathoracic pressure causes a fall in cardiac output due to reduced venous return. In patients with poor left ventricular function, application of PEEP may serve to decrease left ventricular afterload and improve left ventricular performance. A small number of studies also suggest that maintaining airway patency with PEEP may facilitate clearance of secretions [94].

The general physiologic approach to hypoxemia in the absence of confounding factors is to increase mean airway pressure. Elevation in PEEP, the immediate means to this end, has been studied in a variety of multicenter trials. In addition, application of PEEP in patients with chronic obstructive pulmonary disease appears to improve gas flow and mainten airway patency. In the chemical pneumonitis and secretion accumulation, which accompanies smoke inhalation, airway pressure management strategies may do more than optimize oxygenation; gas flows and secretion movement can be favorably affected [89,97].

In the 1980s, intrapulmonary percussion with diffusion of oxygen via subtidal breaths and convective washout of carbon dioxide was introduced by Dr. Forrest Bird. This technology is now marketed as High Frequency Percussive Ventilation (HFPV). The percussive nature of this support enhances clearance of secretions. Cioffi and others have reported improved outcomes with HFPV in patients with inhalation injury for two decades [98-100].

As presently marketed, HFPV machines deliver high frequency subtidal volume breaths followed by a passive exhalation to a baseline preset continuous positive airway pressure. Respiration is time-cycled and pressure limited with frequency, amplitude, inspiratory to expiratory time ratios and waveforms designed to maximize ventilation and perfusion. Pulse frequency of subtidal volume breaths can be varied to assist in providing maximal oxygenation. Typically rates of 500–600 are used initially, but rates can be increased to a maximum of 700–750 if necessary. Amplitude of subtidal volume breaths can also be adjusted to correlate with patient peak inspiratory pressure. Interruption of percussive respiration permits passive CO_2_ elimination. A mandatory respiratory rate is created by variable inspiratory and expiratory times. Initially, a ventilator rate of approximately one-half to two-thirds that of conventional respiration is used for this background pressure. Ventilator variables are subsequently adjusted based on patient response to optimize gas exchange. Conventional ventilator modes are typically used for weaning and extubation. More recent experience with HFPV comes from Hall and coworkers at the University of Texas Southwestern Medical Center. Mortality benefit with HFPV was observed in patients with burns <40% TBSA when comparison was made with individuals receiving a conventional ventilation strategy [98,100,101].

## Medical adjuncts for treatment of smoke inhalation

Current clinical treatment of inhalation injury remains supportive. There has been little recent progress in effective clinical therapies, but there are many promising experimental therapies not yet widely used in patients. Unlike strategies directed specifically as antidotes for products of combustion reviewed above, these interventions address physiologic changes associated with smoke inhalation.

### Beta-agonists

As with other forms of acute lung injury, bronchoconstriction may further worsen already impaired gas exchange in the injured alveoli. The use of inhaled agents targeting beta-adrenoreceptors may help ameliorate this bronchoconstriction. Lange et al. studied nebulized epinephrine in an ovine model of inhalation injury. They divided 15 sheep into three groups: a sham-injury group and two groups with actual inhalation injury, one of which was treated with nebulized saline and the other treated with nebulized epinephrine given every four hours. They found that the nebulized epinephrine group had decreases in airway pressures and increases in PaO_2_/FiO_2_ ratios [102]. In another ovine study by Palmieri et al., continuous nebulized albuterol was given to a group of sheep with a combined burn and inhalation injury and compared to another group receiving nebulized saline. The albuterol cohort had a decrease in airway pressures and an improvement in PaO_2_/FiO_2_ ratio [103].

### Pulmonary blood flow

There are two potential targets for modifying pulmonary blood flow in inhalation injury. The first is diminishing bronchial arterial blood flow and thus, decreasing the flow of systemic inflammatory mediators to the lung. Hamahata et al., again working with a sheep model, surgically ligated the bronchial artery in one group of sheep. They surgically exposed the bronchial artery in the second group but left it intact rather than ligating the artery. They then exposed both groups to a combined burn and smoke inhalation injury. This combined injury increased bronchial blood flow, pulmonary edema, and pulmonary dysfunction in both groups, but all these changes were less severe in the group that had undergone bronchial artery ablation [104]. Building on these initial findings, the same group then exposed the sheep to the burn/smoke inhalation injury first, then used a catheter to inject 70% ethanol into the bronchial artery one hour after injury and compared it to groups with saline injection and with no injury. Again, the injured groups showed markedly worse blood gas analyses and pulmonary mechanics, but those undergoing bronchial artery sclerosis with ethanol had decreased bronchial blood flow and less severe changes in their blood gases and pulmonary mechanics [105].

Another promising modulator of pulmonary blood flow is inhaled nitric oxide (NO). NO is a potent vasodilator that when inhaled will be delivered selectively to ventilated lung and vasodilate the capillaries serving those areas. This results in decreased ventilation/perfusion mismatch, decreased shunting, and decreased pulmonary hypertension [106]. Enkhbataar et al. studied inhaled NO in an ovine model compared to controls not receiving NO. Their model of inhalation injury resulted in increased lung water, increased pulmonary microvascular resistance, and increase pulmonary artery pressures. The NO group had less severe changes in these variables when compared to the control group [107]. Qi et al. studied inhaled NO in a canine model and found that there was also less damage to the myocardium of dogs receiving inhaled NO when compared to a control group. The NO group also had improved cardiac energy metabolism [108].

### Anticoagulants

Significant airway obstruction is one of the hallmarks of inhalation injury. Airway casts are formed by a combination of sloughed epithelial cells, mucus, inflammatory cells, and fibrin. Fibrin in particular has been a target for researchers to attempt to prevent formation of these airway casts.

Enkhbataar et al. used nebulized tissue plasminogen activator (TPA) as a fibrinolytic agent in an experiment with sheep subjected to a combined burn/smoke inhalation injury. They found that TPA-treated sheep had less severe impairment of pulmonary gas exchange, less pulmonary edema, less of an increase in airway pressures, and less airway obstruction than control animals [109]. The same group used a combination of aerosolized heparin and recombinant human antithrombin in another burn and smoke inhalation ovine model. They found that the two agents in combination resulted in better lung compliance, less pulmonary edema, and less airway obstruction than controls. Interestingly, neither agent used alone had the same ameliorating effect [110].

Heparin in combination with N-acetylcysteine gained widespread use after a study by Desai et al. showed decreased mortality in pediatric patients with inhalation injury [111]. However, a subsequent retrospective review by Holt et al. of 150 patients with inhalation injury showed no significant improvement in clinical outcomes in patients treated with inhaled heparin and acetylcysteine [112]. In addition, there has been at least one case report of coagulopathy in a patient receiving aerosolized heparin and acetylcysteine for inhalation injury [113].

Heparin was also combined with the anti-inflammatory agent lisofylline in an ovine model by Tasaki et al. They used three groups of sheep, one receiving nebulized saline only, one getting nebulized heparin only, and the third receiving both nebulized heparin and intravenous lisofylline. The combined heparin/lisofylline group had decreased shunt and less of an increase in alveolar-arterial oxygen tension gradient after a smoke inhalation injury. The heparin-only group did not exhibit these same benefits [114]. The efficacy of aerosolized heparin in the adult burn and inhalation injury population is still unclear.

### Antiinflammatory agents

Reducing the localized inflammatory response after inhalation injury could theoretically decrease the mechanical burden of biomaterials obstructing the airways, as well as decreasing the long-term fibrotic reaction after inhalation injury. There are a number of agents that have been used to reduce inflammation, primarily in animal models.

Thromboxane A2 is an important inflammatory mediator in lung injury, and inhibition of thromboxane synthase has been shown to ameliorate lung injury in both dogs and guinea pigs [115,116]. Westphal et al. used OKY-046 (Ozagrel, 3-[4-(1H-imidazol-1ylmethyl)phenyl]-2E-propanoic acid; Ono Pharmaceutical Co., Osaka, Japan) as a thromboxane synthase inhibitor in a sheep model of smoke inhalation injury. In a group of 16 sheep, eight received the drug and eight received only the drug delivery vehicle. They found that the treatment group had decreased pulmonary thromboxane, and in turn had decreases in pulmonary vascular resistance and less of a decrease in cardiac output [117].

Free oxygen radicals also trigger inflammation during inhalation injury. Scavengers of these reactive oxygen radicals may help attenuate the pathologic inflammatory response to smoke inhalation. Yamamoto et al. used nebulized gamma-tocopherol (in ethanol) in six sheep with severe burns and smoke inhalation and compared them to five sheep with the nebulized ethanol alone. They saw significant improvements in the P:F ratio of the tocopherol group, as well as decreases in pulmonary shunt and airway pressures [118].

The parasympathetic nervous system also contributes to the physiologic response to airway injury by secreting acetylcholine, which acts on muscarinic receptors to constrict smooth muscle in the airways and stimulate activity of submucosal glands. Inhibition of these muscarinic receptors blocks these effects as well as decreasing production of inflammatory cytokines during lung injury [119,120]. Jonkam et al. tested the muscarinic antagonist tiotropium bromide in sheep with no injury, with smoke/inhalation injury, and with smoke/inhalation injury receiving tiotropium bromide. Sheep with a combined burn and inhalation injury showed increases in ventilatory pressures and upper airway obstruction, as well as decreases in P:F ratio. Treatment with this muscarinic receptor antagonist resulted in a lesser degree of pathologic change in all these variables [121].

## Competing interest

The authors declare that there are no competing interests.

## Authors’ contributions

FE and DD performed the literature review and wrote the initial draft of the manuscript. DD conceived of the report and edited and rewrote portions of the manuscript. Both authors read and approved the final manuscript.

## Authors’ information

David J. Dries, MSE, MD, FACS, FCCM, FCCP is the Assistant Medical Director of Surgical Care for HealthPartners Medical Group and Division Head for Surgery at Regions Hospital, the Level I Trauma and Burn Center, in St. Paul, Minnesota, USA. He is also Professor of Surgery, Professor of Anesthesiology and Clinical Adjunct Professor of Emergency Medicine at the University of Minnesota. Dr. Dries also holds the John F. Perry, Jr. Chair of Trauma Surgery at the University of Minnesota.

Frederick W. Endorf, MD, FACS is Staff Surgeon at Regions Hospital, the Level I Trauma and Burn Center, in St. Paul, Minnesota, USA. He is also Clinical Assistant Professor of Surgery at the University of Minnesota.
